# Comparison of remission criteria in a tumour necrosis factor inhibitor treated rheumatoid arthritis longitudinal cohort: patient global health is a confounder

**DOI:** 10.1186/ar4421

**Published:** 2013-12-24

**Authors:** Emese Balogh, Joao Madruga Dias, Carl Orr, Ronan Mullan, Len Harty, Oliver FitzGerald, Phil Gallagher, Miriam Molloy, Eileen O’Flynn, Alexia Kelly, Patricia Minnock, Madeline O’Neill, Louise Moore, Mairead Murray, Ursula Fearon, Douglas J Veale

**Affiliations:** 1 Dublin Academic Medical Centre, Department of Rheumatology, St. Vincent’s University Hospital, Elm Park, Dublin 4, Ireland; 2 Department of Rheumatology, University of Debrecen Medical and Health Science Center, 98 Nagyerdei krt., Debrecen, Hungary

## Abstract

**Introduction:**

Our objectives were to assess the frequency and sustainability of American College of Rheumatology (ACR)/European League against Rheumatism (EULAR) and Disease Activity Score (DAS)28(4v)–C-reactive protein (CRP) remission 12 months after the initiation of tumour necrosis factor inhibitor (TNFi) therapy in a rheumatoid arthritis (RA) cohort.

**Methods:**

Data were collected of 273 biologic naive RA patients at baseline, then 3, 6 and 12 months post-TNFi therapy. Remission status was calculated using DAS28(4v)-CRP <2.6 and ACR/EULAR Boolean criteria. Response was scored using EULAR criteria.

**Results:**

Mean (range) patient age was 59.9 (7.2-85.4) years with disease duration of 13.4 (1.0-52.0) years. Responder status maintained from 3–12 months (86%, 82.4%), laboratory/clinical parameters (erythrocyte sedimentation rate (ESR), CRP, patient global health (PGH), DAS28(4v)-CRP) also showed sustained improvement (*P* < 0.05). DAS28 remission was reached by 102 subjects at 1 year, 27 patients were in Boolean remission, but 75 missed it from the DAS28 remission group. Patients in remission were younger (*P* = 0.041) with lower baseline tender joint count (TJC)28 and PGH than those not in remission (*P* = 0.001, *P* = 0.047). DAS28 remission patients were older (*P* = 0.026) with higher 12 months PGH and subsequently higher DAS28 than Boolean remission patients (*P* < 0.0001). Patients not achieving Boolean remission due to missing one subcriteria most frequently missed PGH ≤1 criteria (79.8%).

**Conclusions:**

Only 10% of this TNFi treated cohort achieved remission according to the new ACR/EULAR criteria, which requires lower disease activity. More stringent criteria may ensure further resolution of disease activity and better longterm radiographic outcome, which supports earlier intervention with biologic therapy in RA.

## Introduction

Early diagnosis and rapid initiation of treatment has become the mission in rheumatoid arthritis (RA).

Several composite indices are used for measuring disease activity, response and remission. The disease activity score based on 28-joint count (DAS28) is a validated method of outcome measures in clinical trials and clinical practice [1]. The DAS28 score, European League Against Rheumatism (EULAR) response criteria, and low disease activity criteria were developed originally using the erythrocyte sedimentation rate (ESR) as an element of the four-variable score (DAS28(4v)-ESR) [2,3]. Later findings highlighted the importance of C-reactive protein (CRP) – being an acute-phase reactant, it might give better estimation of short-term changes in disease activity – and therefore CRP was used in disease assessment as a useful outcome measure in the four-variable DAS28 including CRP (DAS28(4v)-CRP) [4].

Remission criteria using DAS28(4v)-ESR are well known and the most widely used in clinical practice. DAS28-CRP gives a good estimation of DAS28-ESR on a group level [5]. Further recommendations were made on the application of DAS28(4v)-CRP, setting similar cutoff values to the DAS28(4v)-ESR for definitions of disease remission as well as low, moderate or high disease activity [6]. Originally, the remission threshold was set to DAS28(4v)-CRP <2.6, equal to DAS28(4v)-ESR, although some earlier results suggest that DAS-28(4v)-CRP levels and their cutoff values for remission or response are lower [2,6,7]. At present, DAS28 criteria are widely used in formulas either with the ESR or CRP.

More effective drugs and treatment strategies allowed remission to become the treatment target in RA, although even in remission patients can experience radiographic progression [8-10]. Limitations of the previous remission criteria and the ultimate goal of treating to remission urged the collaboration to establish a new composite tool for measuring remission in RA. New RA remission criteria were developed by the American College of Rheumatology (ACR)/EULAR in 2011 on the basis of using data from four clinical trials and need to be validated using different datasets in clinical practice [11]. To be classified as in remission according to these criteria, patients must have swollen joint count out of 28 joints (SJC28), tender joint count out of 28 joints (TJC28), CRP (mg/dl) and patient global health assessment (PGH) on a 0 to 10 visual analogue scale of 1 or less [11].

Previous findings showed that, despite minimal disease activity, patients might not fulfil the ACR/EULAR remission criteria, mainly because these RA patients regularly do not meet the PGH criterion of the provisional ACR/EULAR Boolean-based definition despite a good clinical disease state [12]. Prince and colleagues compared the performance of ACR/EULAR Boolean and Simplified Disease Activity Index (SDAI) remission criteria with DAS28(4v)-ESR criteria and DAS28(4v)-CRP <2.6 or <2.3 criteria [7]. They suggested that overall just a small portion of patients reached and remained in remission in the long term, and fewer patients reached ACR/EULAR remission than any DAS28 remission [7]. The objective of this study was to examine the performance of the ACR/EULAR Boolean and the DAS28(4v)-CRP remission status in a routine clinical setting involving biologic-treated RA patients.

## Methods

### Patient recruitment

Demographic, clinical and laboratory data of a RA cohort (*n* = 273), prior to commencing tumour necrosis factor-alpha inhibitor (TNFi) therapy, were collected in the Distiller database at routine clinics in Dublin. All data collection was compliant with the Helsinki declaration and good clinical practise; ethical approval was not required by the St. Vincent’s University Hospital Research Ethics committee. All patients have been diagnosed with RA according to the 1987 classification criteria and were due to start biological treatment for persistent disease activity; no consent was needed [13]. Data were collected at four timepoints: at baseline (0), and then 3, 6 and 12 months after initiating TNFi therapy.

### Data collection

Demographic data included patient age, sex and disease duration (years). The following clinical and laboratory parameters have also been recorded at all timepoints: TJC28, SJC28, PGH on a 0 to 10 visual analogue scale, levels of CRP, ESR and DAS28(4v)-CRP. We did not stratify patients by therapy, and nor did we stage by Steinbrocker functional class.

### Scoring response, low disease activity and remission

Response to treatment was scored with EULAR response criteria in three categories: good response, moderate response, and nonresponse [14]. Remission status was calculated using both DAS28(4v)-CRP remission criteria and ACR/EULAR Boolean criteria [11-15]. Patients with DAS28(4v)-CRP <2.6 fulfilled DAS28 remission. ACR/EULAR Boolean remission was defined by the following: TJC28 ≤1, SJC28 ≤1, PGH ≤1, and CRP (mg/dl) ≤1. Low disease activity was defined by 2.6 ≤ DAS28(4v)-CRP ≤3.2 [6,14,16].

### Statistical analysis

Statistical analysis of nonparametric variables was performed with SPSS software (v20). Comparison of DAS28(4v)-CRP and DAS28(4v)-ESR remission criteria was performed using the κ formula with the interpretation of Landis and Koch for κ values, where <0 indicates no agreement, 0 to 0.2 slight, 0.21 to 0.40 fair, 0.41 to 0.60 moderate, 0.61 to 0.80 substantial, and 0.81 to 1.0 almost perfect agreement [5]. Improvement of clinical and laboratory data (TJC28, SJC28, PGH, CRP, ESR) was analysed by the Wilcoxon signed-rank test. Baseline data are presented as mean ± standard deviation or percentages (%) calculated by the chi-square test. The Mann–Whitney U test was applied for calculations between independent nonparametric variables. All *P* values are two-sided and *P* <0.05 was considered statistically significant.

## Results

### Demographic data

Mean (range) patient age was 59.9 (7.2 to 85.4) with mean (range) disease duration of 13.4 (1.0 to 52.0) years, and mean ± standard deviation DAS28(4v)-CRP score of 5.33 ± 1.07. The female:male ratio was 3:1, and 70.2% of patients were rheumatoid factor-positive and 75.2% were anti-cyclic citrullinated peptide positive. Baseline demographics are detailed in Table 1. At baseline none of the patients were in Boolean or DAS28 remission and due to their high disease activity anti-TNF treatment was commenced.

**Table 1:** Baseline demographic and clinical data of rheumatoid arthritis patients in different 12-month remission statuses

	**Whole cohort (*n*** **= 273)**	**DAS28 remission (*n*** **= 102)**	**Only DAS28 remission (*n*** **= 75)**	**DAS28 + Boolean remission (*n*** **= 27)**	**Not in any remission (*n*** **= 171)**
Age (years)	59.89 (7 to 85)	57.12 (7 to 85)	59.17 (26 to 85)	51.43 (7 to 75)	61.53 (30 to 86)
Disease duration (years)	13.43 (1 to 52)	11.1 (1 to 40)	12,23 (1 to 40)	7.91 (1 to 21)	14.68 (1 to 52)
Female (%)	74.7	68.6	66.7	74.1	78.4
RF positivity (%)	70.2	69.7	71.2	65.4	70.5
Anti-CCP positivity (%)	75.2	69.4	65.7	78.6	79.7
DAS28(4v)-CRP	5.33 ± 1.07	5.15 ± 1.1	5.18 ± 1.16	5.08 ± 0.9	5.44 ± 1.05
TJC28	10.24 ± 6.86	8.71 ± 6.83	9.19 ± 7.34	7.37 ± 5.05	11.15 ± 6.73
SJC28	9.86 ± 6.20	9.6 ± 6.25	9.73 ± 6.57	9.22 ± 5.36	10.02 ± 6.19
CRP (mg/dl)	2.76 ± 2.93	3.16 ± 3.14	3.23 ± 3.12	2.93 ± 3.26	2.53 ± 2.78
PGH	5.92 ± 2.28	5.6 ± 2.29	5.57 ± 2.33	5.68 ± 2.22	6.12 ± 2.26

Data presented as mean (range), mean ± standard deviation or percentage. DAS28, disease activity score based on 28-joint count; RF, rheumatoid factor; anti-CCP, anti-cyclic-citrullinated peptide antibody; DAS28(4v)-CRP, disease activity score based on 28-joint count, C-reactive protein and patient global health; TJC28, tender joint count out of 28 joints; SJC28, swollen joint count out of 28 joints; CRP, C-reactive protein; PGH, patient global health.

### Applying DAS28(4v)-CRP and DAS28(4v)-ESR remission criteria to the cohort (remission <2.6)

We compared remission and response rates at different timepoints with these two criteria and found at least moderate agreement between the two groups at any timepoint (κ value >0.556, *P* <0.0001 at any timepoint); hence we decided to perform further calculations with the DAS28(4v)-CRP formula (Table 2).

**Table 2:** Agreement of remission and response rates calculated with DAS28(4v)-ESR and DAS28(4v)-CRP at different timepoints after initiating biological therapy

	**κ value**
Remission – 3 months	0.689 (*P* <0.0001)
Remission – 6 months	0.617 (*P* <0.0001)
Remission – 12 months	0.717 (*P* <0.0001)
Response – 3 months	0.556 (*P* <0.0001)
Response – 6 months	0.731 (*P* <0.0001)
Response – 12 months	0.725 (*P* <0.0001)

κ value according to Landis and Koch interpretation. DAS28(4v)-ESR, disease activity score based on 28-joint count, erythrocyte sedimentation rate and patient global health, DAS28(4v)-CRP, disease activity score based on 28-joint count, C-reactive protein and patient global health.

### Patients in remission

There was no significant difference between the number of patients achieving remission with DAS28(4v) or three-variable disease activity score based on 28 swollen and tender joint count, and C-reactive protein (DAS(3v)) criteria (*n* = 102 vs. *n* = 115) in this cohort at 1 year, so we made further calculations with DAS28(4v).

All ACR/EULAR Boolean remission patients were in DAS28 remission at all timepoints. Baseline demographic and clinical data for remission groups are presented in Table 1, and 1-year remission demographic and clinical data are presented in Table 3.

**Table 3:** Twelve-month demographic and clinical data of rheumatoid arthritis patients in different remission statuses

	**DAS28 remission (*n*** **= 102)**	**Only DAS28 remission (*n*** **= 75)**	**DAS28 + Boolean remission (*n*** **= 27)**	**Not in any remission (*n*** **= 171)**
DAS28(4v)-CRP	1.99 ± 0.31	2.08 ± 0.28	1.75 ± 0.27	3.97 ± 1.12
TJC28	0.17 ± 0.45	0.17 ± 0.48	0.15 ± 0.36	4.92 ± 5.39
SJC28	0.41 ± 1.17	0.53 ± 1.34	0.07 ± 0.27	4.05 ± 4.66
CRP (mg/dl)	0.53 ± 0.61	0.56 ± 0.71	0.43 ± 0.14	1.28 ± 2.84
PGH	2.08 ± 1.56	2.58 ± 1.52	0.7 ± 0.46	4.99 ± 2.39
Low disease activity (%)	100	100	100	28.1
Responder (%)	100	100	100	71.9
Good response (%)	94.1	92	100	24.6
Moderate response (%)	5.9	8	0	47.4
Nonresponse (%)	0	0	0	28.1

Data presented as mean ± standard deviation or percentage. DAS28, disease activity score based on 28-joint count; DAS28(4v)-CRP, disease activity score based on 28-joint count, C-reactive protein and patient global health; TJC28, tender joint count out of 28 joints; SJC28, swollen joint count out of 28 joints; CRP, C-reactive protein; PGH, patient global health.

From the cohort of 273 patients, 102 subjects (37%) were in DAS28 remission, 75 (27%) patients were in DAS28 remission alone and 27 (10%) were in both DAS-28 and Boolean remission at 12 months. Boolean and DAS28 remission rates were compared with no-remission. Patients in DAS28 remission were significantly younger (*P* = 0.041) and had lower baseline TJC28 and PGH scores than their nonremission counterparts (*P* = 0.001, *P* = 0.047). They also had significantly lower 12-month disease activity (TJC28, SJC28, CRP, PGH, DAS28(4v)-CRP: all *P* <0.0001) (Tables 1 and 3). Boolean remission patients were younger (*P* = 0.003) with lower baseline TJC28 (*P* = 0.028) and 12-month disease activity (TJC28, SJC28, CRP, DAS28(4v)-CRP: all *P* <0.025) than nonremission patients (Tables 1 and 3).

**Figure 1:**
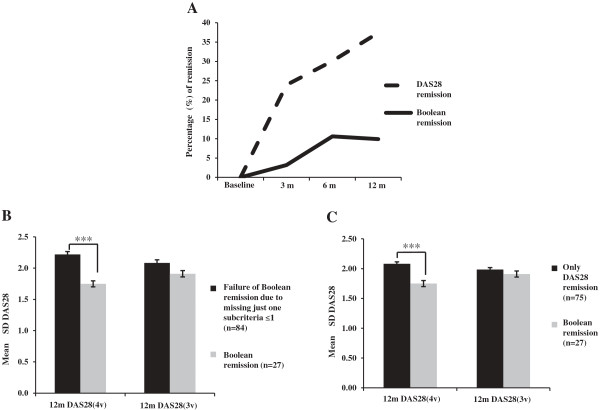
**Characteristics of disease activity score based on 28 joints and Boolean remission groups. (A)** Percentage of patients in disease activity score based on 28 joints (DAS28) remission versus Boolean remission. 3 m, 6 m and 12 m: 3 months, 6 months and 12 months post tumour necrosis factor inhibitor therapy. **(B)** The 1-year four-variable disease activity score (DAS28(4v)) was higher for patients who failed Boolean remission due to just one subcriterion being ≤1, but disease activity did not differ from Boolean remission calculated with the three-variable formula (DAS28(3v)). This explains that significant differences in disease activity resulted mainly in higher patient global health. **(C)** The DAS28(4v) was higher in the only DAS28 remission group, but disease activity did not differ from Boolean remission patients calculating with the DAS28(3v). This explains that significant differences in disease activity resulted mainly in higher patient global health. ***P <0.0001. SD, standard deviation.

Significantly fewer patients reached Boolean remission than DAS28 remission (*P* <0.0001) (Figure 1A) at all timepoints, and therefore we investigated the reasons for differences in disease activity with comparing Boolean remission group versus the part of DAS28 remission group who missed ACR/EULAR remission (only DAS28 remission group). We found that Boolean remission patients were younger (*P* = 0.026) and had lower 12-month DAS28(4v)-CRP and PGH values than only DAS28 remission patients (both *P* <0.0001) (Tables 1 and 3).

The maximum level of disease activity for a Boolean remission patient in our cohort was 2.52 calculated by DAS28. Because Boolean criteria do not allow such variability in the subcriteria scores as does DAS28, at any timepoint fewer patients fulfil Boolean remission due to the originally more stringent disease activity criteria. These data suggest that Boolean remission criteria are originally stricter in defining remission than the DAS28 criteria. In view of the latter, Boolean remission patients are more likely to be better responders for biological therapy than DAS28 remission patients.

### Patient global health as a limiting factor of Boolean remission status

We identified patients who fulfil most Boolean remission criteria with the exception of one variable (*n* = 84) and concluded that higher PGH has a greater impact on remission status than other variables, with an effect towards nonremission. Even though there were three remission criteria fulfilled, 67 patients (80%) did not reach remission due to residual disease activity of PGH >1, another eight patients (9%) due to SJC >1, four patients (5%) due to TJC >1 and five patients (6%) due to CRP >1. These results are in concordance with the findings of Studenic and colleagues in 2012 about the limiting characteristics of PGH [17].

For those patients missing Boolean remission failing just one subcriterion at 1 year (*n* = 84), 12-month DAS28(4v)-CRP scores were higher (*P* <0.0001) than scores of patients reaching Boolean remission. Although 12-month SJC28 was also higher for the failing subgroup (*P* = 0.027), this difference in disease activity resulted from mainly higher 12-month PGH scores of the failing patients (*P* <0.0001) because DAS28(3v)-CRP calculation did not reveal significant differences in the disease activity between the two subcategories (*P* >0.05) (Figure 1B). Higher PGH has an important role in excluding patients from Boolean remission creating higher disease activity, and consequently Boolean remission has the advantage to exclude residual disease activity resulting from high scores of PGH in the definition of remission.

### Why do some of the DAS28 remission patients miss Boolean remission?

We compared the 12-month disease activity of only DAS28 remission patients and their Boolean remission counterparts. In spite of the higher DAS28(4v)-CRP values at 12 months for the only DAS28 remission group (*P* <0.0001), there were no differences between DAS28(3v)-CRP scores between the two categories (*P* = 0.26). Higher PGH values were thereby also proved to contribute to DAS28(4v)-CRP differences between remission categories (Figure 1C). These data suggest that DAS28 remission criteria allow much greater residual PGH-related disease activity for individuals being in remission.

### Seropositivity and remission status

There was no difference in remission rates of rheumatoid factor or anti-cyclic citrullinated peptide positive/negative patients according to either remission criteria; however, DAS28 remission allowed more seropositive and seronegative patients into remission than Boolean criteria (*P* <0.05).

### Response to treatment

Clinical and laboratory data (TJC28, SJC28, PGH, CRP, ESR, DAS28(4v)-CRP) showed significant sustained improvement during biological therapy in each remission group from 3 to 12 months (all *P* <0.05), but nonremission patients showed poorer improvement of disease activity during treatment. The majority of patients were responders and response rates showed a shift from moderate towards good response by 12 months, and the low disease activity status similarly improved (Table 4). Boolean remission patients more frequently showed good response to therapy than DAS28 remission patients, but most of the nonremission patients also reached at least moderate response during TNFi therapy (Table 4). Both DAS28 and Boolean remission statuses showed increasing remission rates from 3 to 6 months, and then DAS28 remission further improved and Boolean remission stagnated (Table 4).

**Table 4:** EULAR response, low disease activity and remission rates during TNF inhibitor treatment of the rheumatoid arthritis cohort

	**3 months**	**6 months**	**12 months**
Good response (%)	39.5	45.8	50.5
Moderate response (%)	49.5	39.8	31.9
Nonresponse (%)	14	14.4	17.6
DAS28(4v)-CRP <3.2 (%)	42.3	50.9	54.9
Low disease activity (%)	18.5	20.5	17.6
DAS28 remission	53 (23.9%)	65 (30.1%)	102 (37.4%)
Boolean remission	7 (3.2%)	23 (10.6%)	27 (9.9%)

EULAR, European League Against Rheumatism, TNF, tumour necrosis factor; DAS28(4v)-CRP, disease activity score based on joints using four variables and including C-reactive protein as the inflammatory marker; DAS28, disease activity score based on 28 joints.

### Composite model for predicting Boolean remission versus DAS28 remission statuses

Predictive factors for the different remission status were investigated by binary logistic regression analysis, which revealed that younger age and lower baseline TJC28 predict for both DAS28 and Boolean remission statuses compared with nonremission. Younger age also predicts Boolean remission versus only DAS28 remission at 12 months (Table 5).

**Table 5:** Binary logistic regression analysis for the prediction of different remission statuses

	**DAS28 remission versus nonremission**		**Boolean remission versus nonremission**		**Boolean versus only DAS28 remission**	
	**OR**	***P*** **value**	**OR**	***P*** **value**	**OR**	***P*** **value**
Age	0.968	0.004	0.942	<0.0001	0.956	0.014
Baseline TJC28	0.946	0.018	0.910	0.031	–	–

Analyses were controlled for baseline levels of disease activity components: C-reactive protein (mg/dl), tender joint count out of 28 joints (TJC28), swollen joint count out of 28 joints, and patient global health. DAS28, disease activity score in 28 joints. *P* <0.05 indicates the association of the variable with different remission statuses. Odds ratios (ORs) explain the probability of reaching different remission statuses with higher age and baseline TJC28.

## Discussion

Remission-orientated treatment is the principal target either with disease-modifying antirheumatic drugs or biological therapy in RA [18,19]. Several composite indices have been introduced to evaluate disease activity in rheumatology practice (DAS28, SDAI, Clinical Disease Activity Index) [20]. Increasing postulation for early diagnosis and aggressive treatment has created a demand for more stringent measures of response, especially remission in disease development. In addition, it has been suggested that it is possible to predict early response to TNFi therapies using a mathematical model [21]. Previous remission criteria have been used in daily practice and clinical trials, of which DAS28 remission criteria are the most commonly used and reliable in clinical practice [22]. The need for other stringent remission criteria created a collaboration by ACR, EULAR and the Committee of Outcome Measures in Rheumatology Initiative (OMERACT) group to work out the new ACR/EULAR remission criteria in 2011 [11]. In this observational cohort we examined the frequency and sustainability of remission according to the new criteria and the DAS28(4v)-CRP-based criteria.

We followed a biologic-naive RA group being unresponsive for different disease-modifying agents (disease-modifying antirheumatic drugs), having high disease activity and requiring the initiation of biological therapy. The examined patients were started on TNFi therapy that is unique in terms of the homogenicity of the RA group. Investigations were made in a clinical setting.

Boolean remission criteria allow fewer patients into remission than DAS28(4v)-CRP criteria, and hence the highest possible score for any subcriteria is ≤1; however, DAS28 allows wide varieties also in TJC, SJC, CRP and PGH. ACR/EULAR remission criteria are more stringent in terms of allowing fewer patients into remission, and in the view of these results fewer patients can reach this requirement due to higher TJC28, SJC28, CRP or PGH values at any timepoint. For the same reason, Boolean remission patients were better responders on a group basis than DAS28 remission patients. Recently de Punder and colleagues found that anti-tumour necrosis factor-treated patients who score 1 on every item of the ACR/EULAR remission criteria had a DAS28(4v)-ESR higher (2.8) than the cutoff point for their DAS28(4v)-ESR remission (<2.6) [23]. Hirabayashi and Ishii revealed that the DAS28-ESR cutoff point necessary to predict remission was <1.54 under the new criteria in clinical settings for tocilizumab-treated patients; a DAS28-ESR cutoff point <2.0 was previously examined as a candidate definition of remission by ACR/EULAR [11,24].

Several published reports have examined the effect of PGH on ACR/EULAR criteria for clinical trials, and emphasise that missing the PGH criteria by ≤1 frequently leaves patients in a nonremission status. Otherwise these patients demonstrate a good clinical disease activity [12]. Similar observations were also confirmed in a solely clinical setting of the ACR/EULAR criteria, neglecting CRP from the definition of remission [6]. We present the first PGH data on a biologic naive cohort starting TNFi treatment. In patients missing Boolean remission due to one subcriterion, PGH was found to be the strongest contributory factor raising disease activity above the cutoff in this cohort. In accordance with this, DAS28 remission patients missing Boolean remission status have higher post-treatment disease activity, primarily due to increased PGH. Previous findings suggest that the assessment of PGH may raise difficulties in defining remission in clinical practice. Originally, the ACR/EULAR remission criteria were developed for clinical trials. The literature reflects that clinical trial conditions are different from routine clinical practice and that patients may score PGH differently depending on their original disease activity [12]. Modification of PGH subcriteria to PGH ≤2 has been proposed to allow more patients to reach ACR/EULAR remission [25]. The limitation in clinical application of the new remission criteria is that patients may score a high PGH for reasons other than RA disease activity, raising the opportunity for overtreatment; in contrast, minimisation of latent disease activity may ensure better long-term radiographic outcomes.

We confirmed that despite remission rates being lower in Boolean remission status, the majority of patients gave at least moderate response to therapy and good response improved from 3 to 12 months. One-fifth of our patients presented low disease activity post treatment at any timepoint and both the DAS28 and Boolean remission statuses improved from 3 to 12 months of therapy.

The feasibility of more stringent remission criteria hides inherent challenges as age, comorbidities and nondisease-related factors may significantly influence measurement of remission. In this study we confirmed that younger age and lower baseline TJC28 were predictors for both DAS28 and Boolean remission status compared with nonremission, which is consistent with previous findings that gave impact for these predictors in DAS28 remission [26]. Age has a further impact on remission status, younger patients being more likely to reach Boolean remission compared with DAS28 remission, which supports earlier aggressive treatment as it offers a higher probability of sustained remission.

The drawbacks to our study are that calculations with the Clinical Disease Activity Index and SDAI were not calculated due to lack of data. We did not compare remission criteria in the case of patients on solely disease-modifying agents (disease-modifying antirheumatic drugs) and we did not analyse according to different TNFi therapies, although most studies to date have failed to show differences in efficacy across the class. Our observational study did not extend to data analysis on the predictive value of remission criteria for further synovitis, bone marrow oedema or radiographic progression. There is evidence that a longer period in remission results in less radiographic progression [10]. Additionally, several studies confirm that subclinical disease activity, and possibly further radiological progression, may be detectable despite clinical remission, and applying stricter remission criteria should guard against this happening [27]. Zhang and colleagues examined the radiologic and functional outcome of RA patients 1 year after treatment initiation and found that Boolean and SDAI/Clinical Disease Activity Index definitions made better prediction for good outcomes than DAS-based definitions [28]. Lillegraven and colleagues first evaluated the relationship between the time in remission and radiographic joint damage comparing ACR/EULAR with DAS28-CRP-based remission on a clinical observational RA cohort. Their findings suggest that the Boolean-based definition has the highest, and the DAS28-CRP-based definition has the lowest, likelihood ratio for good radiographic outcome, therefore verifying the validity of new remission criteria in clinical settings [10]. Sakellariou and colleagues also confirmed the higher probability of ultrasound-detected synovitis for DAS28 remission than ACR/EULAR remission [29]. However, we did not examine ultrasound/magnetic resonance imaging-detected synovitis. Existing subclinical disease activity in clinical remission establishes the demand for further observational studies in the state of remission to introduce new recommendations including imaging follow-up strategies. Identifying predictors of remission will help to achieve a better radiographic and functional outcome [30].

## Conclusions

Remission remains the ideal therapeutic target. DAS28 criteria define the remission state allowing residual disease activity. High PGH scores do have an impact on defining remission status using the new Boolean criteria. The new criteria are more stringent in defining remission, suggesting they may lead to better long-term radiographic outcomes. Patients at a younger age are more likely to reach Boolean remission compared with DAS28 remission, emphasising the importance of early diagnosis and appropriate treatment in RA. It will be interesting to examine the utility of the new ACR/EULAR remission criteria in long-term follow-up studies focusing on radiographic outcome.

## Abbreviations

ACR: American College of Rheumatology; CRP: C-reactive protein; DAS28(3v)-CRP: Three-variable disease activity score based on 28 swollen and tender joint count, and C-reactive protein; DAS28(4v)-CRP: Four-variable disease activity score based on 28 swollen and tender joint count, C-reactive protein and patient global health score; DAS28(4v)-ESR: Four-variable disease activity score based on 28 swollen and tender joint count, erythrocyte sedimentation rate and patient global health score; DAS28: Disease activity score based on 28-joint count; ESR: Erythrocyte sedimentation rate; EULAR: European League Against Rheumatism; PGH: Patient global health; RA: Rheumatoid arthritis; SDAI: Simplified disease activity index; SJC28: Swollen joint count out of 28 joints; TJC28: Tender joint count out of 28 joints; TNFi: Tumour necrosis factor inhibitor.

## Competing interests

The authors declare that they have no competing interests.

## Authors’ contributions

All authors contributed to the acquisition and interpretation of data, and drafting the manuscript. In addition, EB, JMD, UF and DJV made substantial contributions to the study conception, data analysis and revision of manuscript. CO, RM and LH participated in patient recruitment and data analysis. AK, OF, PG, MMo, EO’F, PM, LM, MO’N and MMu contributed to patient recruitment and data capturing. All authors read and approved the final manuscript.

## Authors’ information

EB and JMD shared first authorship.
